# Supplementary material for: Learning curve and surgical outcome of robotic assisted colorectal surgery with ERAS program
Source: Sci Rep. 2022 Nov 29;12:20566. doi: 10.1038/s41598-022-24665-w (PMC9709162; doi:10.1038/s41598-022-24665-w)
Supplement: Supplementary file 1 — Supplementary Information 1. [file 41598_2022_24665_MOESM1_ESM.zip › LSC Raw data-Ñ[▒K/122/N_ñw╝╨░O▒Kñσ.pdf]

W52 / W52

臺中榮民總醫院
手術室護理記錄

[redacted]

基本資料

- 室別： 開刀房-06室，手術科別： CRS，手術類別： 常規，日期： 2021/03/18
- 進入時間： 1130 ； 麻醉開始時間： 1135 ； Time Out時間： 1235 ； 劃刀時間： 1236 ； 手術結束時間： 1525 送出時間： 1550 ；
- 麻醉方式： GE
  診斷： Malignant tumor of hepatic flexure
  手術名稱： Laparoscopic right colectomy and anastomosis
  手術局部用藥： nil
  術前預防性抗生素：
  - 抗生素名稱一： Veterin 1 gm IVD由麻醉人員給予
  - 時間： 1225
  - 記錄者： 何宛臻

  ，追加預防性抗生素：
- 手術醫師： 蔣鋒帆，林陳彥，黃色霖，楊立宇，劉佳樺
- 麻醉醫師： VS 王賜洲
-
  - 刷手護士A ： 廖子晴，時間： 1130，接替刷手一： 何育晴，時間： 1200，接替刷手二： 何佳青，時間： 1300
  - 流動護士A ： 何佳青，時間： 1130，接替流動一： 廖子晴，時間： 1230
- 更多接替及流動護士： 接替刷手三： 李郁萱，時間： 1500，接替流動三： 何宛臻，時間： 1500

皮膚狀況

A-V Shunt： 無
術前： 完整
記錄者： 上次簽章: 廖子晴
術後： 完整
記錄者： 何宛臻

手術準備

手術臥式 單一手術臥式

-
  - 手術臥式： 仰臥
  - 使用附件： 溫毯 38 ℃，頭圈
  - 皮膚準備： 10%Aq Betadine，Hibiscrub，2% Chlorhexidine
  - 電燒： 使用電燒: 電極片位置： 其它： 迴路電擊墊，記錄者： 上次簽章: 廖子晴

止血帶T： 無

植入物

- 植入物名稱/號碼/數量/部位： Ethicon GIA 75mm x 2釘/ A-colon.

, 記錄者： 上次簽章: 廖子晴

標本送檢

- 細菌培養 無
- 細胞學 無
- 冰凍切片 無
- 病理標本
  組織名稱一： A-colon
- 截肢 無

標本交付 標本： 1 袋，標本： 0 盒，標本： 0 瓶，細胞學： 0 管，Culture Tube： 0 管，被交付醫師： CR 林陳彥，

- 記錄者： 何宛臻

- 時間： 1535

傷口術後敷料

傷口敷料： 傷口敷料： 無

- 請於圖上標示部位：

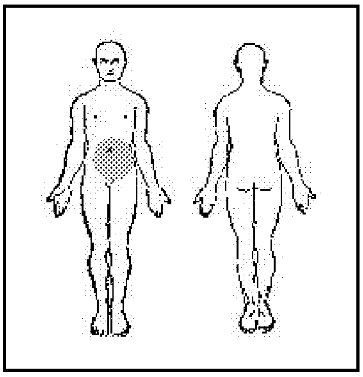

- 傷口敷料： 軟性敷料

導管

導管	天數	導管資訊	插入日期	插入時間	更換日期	拔除日期	拔除時間	拔除原因	預計拔除/更換	預計拔除/更換日期
Epidural PCA(1)	1	部位:脊髓;	2021/03/18	1115						
Foley(1)	1	號碼:16 Fr.; 部位:膀胱;	2021/03/18	1200						
A-line (1)	1	號碼:22 Ga; 部位:左手;	2021/03/18	1150						
CVC(1)	1	號碼:8 Fr; 深度:15cm; 部位:jugular (右); 象限:RU;	2021/03/18	1205						
Sump NG Tube(1)	1	號碼:16 Fr.; 部位:胃;	2021/03/18	1210						

用物計數

- 手術中出血量： 少量 c.c.
- 手術中尿量： 250 c.c.

用物計數： 多切口同時縫合

- 1.器械： 正確 , 2.縫針： 正確 , 3.刀片： 正確 , 4.紗布： 正確 , 5.腹部墊： 未使用 , 6.紗球： 未使用 , 7.PEANUTS ： 未使用 , 8.紗條： 未使用 , 9.絲帶： 未使用 , 10.針頭： 未使用 , 11.Vessel loop： 未使用 , 12.Boots： 未使用 , 13.Jelco： 未使用 , 14.棉條： 未使用 ,
    - 第一次：
        - 刷手護士 ： 何佳青
        - 流動護士： 上次簽章: 廖子晴
        - 時間： 1445

    ,
    - 第二次：
        - 刷手護士 ： 李郁萱
        - 流動護士： 何宛臻
        - 時間： 1500

    ,
    - 第三次：
        - 刷手護士 ： 李郁萱
        - 流動護士： 何宛臻
        - 時間： 1510

備註

備註： 腹腔鏡no.5

- 記錄者： 上次簽章: 廖子晴
- 時間： 1235

填寫護理人員 何宛臻

正式摘要

		W52 / W52			

doctorRank:B
